# Targeting mitochondrial fusion and fission proteins for cardioprotection

**DOI:** 10.1111/jcmm.15384

**Published:** 2020-05-14

**Authors:** Sauri Hernandez‐Resendiz, Fabrice Prunier, Henrique Girao, Gerald Dorn, Derek J. Hausenloy, EU‐CARDIOPROTECTION COST Action (CA16225)

**Affiliations:** ^1^ National Heart Research Institute Singapore National Heart Centre Singapore Singapore Singapore; ^2^ Cardiovascular & Metabolic Disorders Program Duke‐National University of Singapore Medical School Singapore Singapore; ^3^ Centro de Biotecnologia‐FEMSA Tecnologico de Monterrey Nuevo Leon Mexico; ^4^ Institut MITOVASC CNRS UMR 6015 INSERM U1083 University Hospital Center of Angers University of Angers Angers France; ^5^ Faculty of Medicine Coimbra Institute for Clinical and Biomedical Research (iCBR) University of Coimbra Portugal; ^6^ Center for Innovative Biomedicine and Biotechnology University of Coimbra Coimbra Portugal; ^7^ Clinical Academic Centre of Coimbra (CACC) Coimbra Portugal; ^8^ Department of Internal Medicine Center for Pharmacogenomics Washington University School of Medicine St. Louis MO USA; ^9^ Yong Loo Lin School of Medicine National University Singapore Singapore Singapore; ^10^ The Hatter Cardiovascular Institute University College London London UK; ^11^ Cardiovascular Research Center College of Medical and Health Sciences Asia University Taichung Taiwan

**Keywords:** acute myocardial ischaemia/reperfusion injury, cardioprotection, mitochondrial morphology, mitochondrial unfolded protein response, mitophagy cardioprotection

## Abstract

New treatments are needed to protect the myocardium against the detrimental effects of acute ischaemia/reperfusion (IR) injury following an acute myocardial infarction (AMI), in order to limit myocardial infarct (MI) size, preserve cardiac function and prevent the onset of heart failure (HF). Given the critical role of mitochondria in energy production for cardiac contractile function, prevention of mitochondrial dysfunction during acute myocardial IRI may provide novel cardioprotective strategies. In this regard, the mitochondrial fusion and fissions proteins, which regulate changes in mitochondrial morphology, are known to impact on mitochondrial quality control by modulating mitochondrial biogenesis, mitophagy and the mitochondrial unfolded protein response. In this article, we review how targeting these inter‐related processes may provide novel treatment targets and new therapeutic strategies for reducing MI size, preventing the onset of HF following AMI.

## 1 INTRODUCTION

Acute myocardial infarction (AMI) and the heart failure (HF) that can follow are among the leading causes of death and disability worldwide. Although mortality following AMI is on the decline, the prevalence and severity of HF is rising. Therefore, new treatments are required to protect the myocardium against the detrimental effects of acute ischaemia/reperfusion (IR) injury in order to reduce myocardial infarct (MI) size, preserve left ventricular (LV) function and prevent the onset of HF.^1^ Maintenance of healthy mitochondria is of critical importance, given the high energy demands required for normal cardiac contractile function.^2^ Under conditions of energy stress such as experienced during acute myocardial ischaemia/reperfusion injury (IRI) following AMI, damaged mitochondria generate less ATP and produce reactive oxygen species (ROS) that are detrimental to cell survival. As such, new therapies that are able to preserve mitochondrial function during acute myocardial IRI may provide novel strategies for cardioprotection.^2^ The mitochondrial fusion and fission proteins have been shown to play critical roles in several processes related to mitochondrial quality control including mitochondrial biogenesis, mitophagy and the unfolded protein response (UPR). Mitochondrial fission is required to selectively remove damaged mitochondria by mitophagy (which are then replaced by mitochondrial biogenesis), and mitochondrial fusion allows fragmented mitochondria which are still viable to re‐enter the mitochondrial network (reviewed in Refs 2, 3, 4). The mitochondrial UPR is a cytoprotective signalling pathway triggered by the mitochondrial accumulation of toxic unfolded proteins under conditions of cellular stress.^5^ In this article, we review how targeting these inter‐related processes related to mitochondrial quality control may provide novel treatment targets and new therapeutic strategies for reducing MI size and preventing HF following AMI.

## 2 MITOCHONDRIAL MORPHOLOGY

**FIGURE 1:**
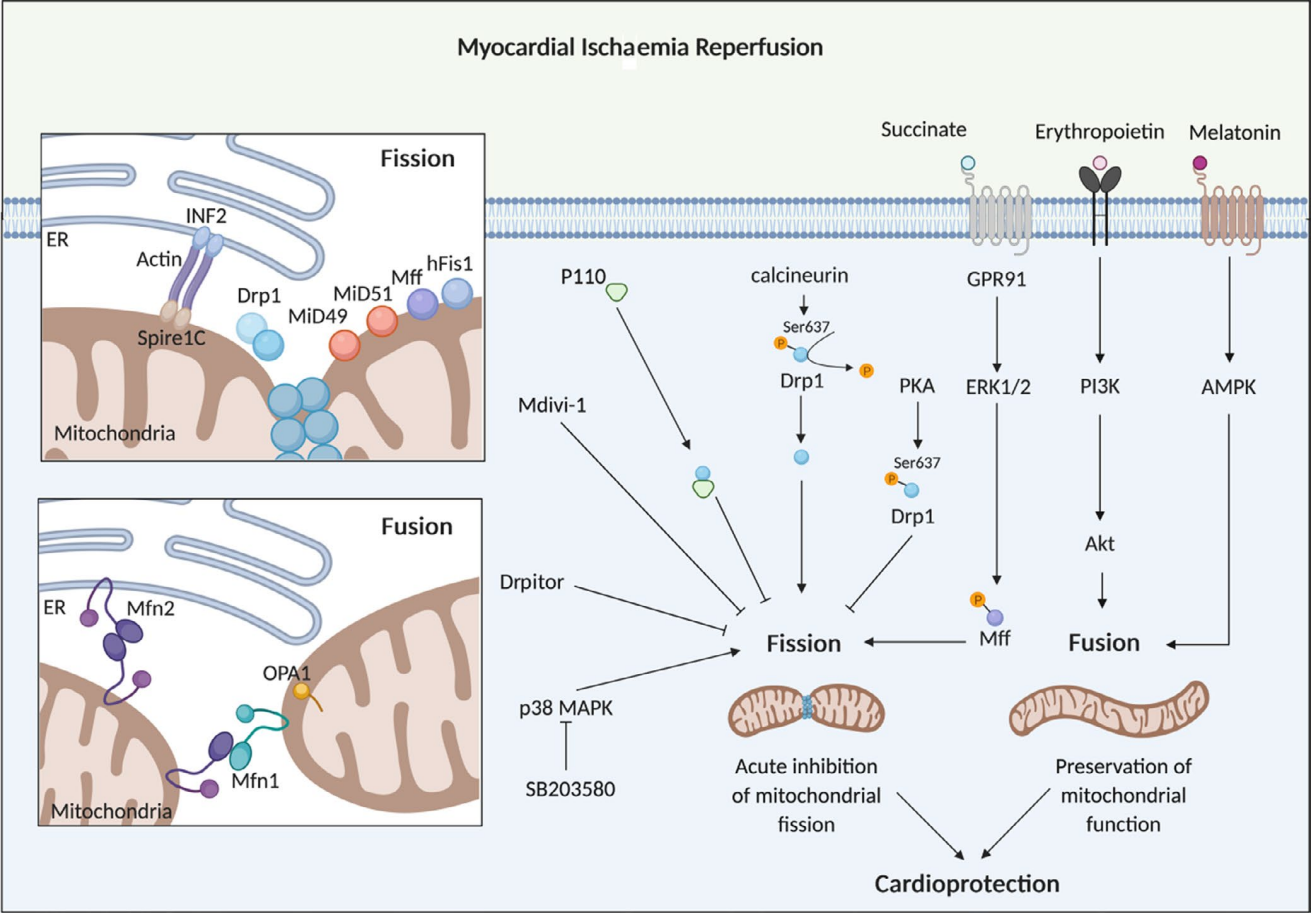
Mitochondrial fission and fusion proteins as targets for cardioprotection. Mitochondrial fission induced by acute myocardial ischaemia/reperfusion injury can be targeted by mdivi‐1 and Drpitor, pharmacological inhibitors of Drp1 and P110, a peptide inhibitor of the interaction between Drp1 and hFis1 to confer cardioprotection. A number of other factors (such as SB203580, PKA activators, succinate, erythropoietin and melatonin) have also been shown to confer cardioprotection by targeting the fusion and fission proteins. Inset upper box: This scheme shows the mitochondrial fission machinery, comprising Drp1 and its outer mitochondrial membrane receptors, MiD49/MiD51, Mff and hFis1. Pre‐constriction by the endoplasmic reticulum (ER) via INF2, actin and Spire1C initiates the Drp1‐driven mitochondrial fission process. Inset lower box: This scheme shows the pleiotropic non‐fusion effect of Mfn2 in tethering mitochondria to the ER

Mitochondria are dynamic organelles, constantly changing their morphology or shape between a fragmented disconnected phenotype by undergoing fission, and an elongated interconnected morphology by undergoing fusion, processes which are coordinated by the mitochondrial fission and fusion proteins, respectively (Figure 1) (reviewed in Refs 2, 3, 4). Mitochondrial fission is essential for cell division and is required to remove damaged mitochondria by mitophagy. In contrast, mitochondrial fusion enables the replenishment of damaged mitochondrial DNA and facilitates intracellular energy distribution. A brief overview of these mitochondrial shaping proteins is given here, with the main focus being on their roles in acute myocardial IRI, and as potential therapeutic targets for acute cardioprotection.

Mitochondrial fission involves the division of a single mitochondrion into 2 individual fragmented and disconnected mitochondria by the fission proteins, dynamin‐related protein 1 (Drp1),^6^ human fission protein 1 (hFis1),^7^ mitochondrial fission factor (Mff)^8^ and the mitochondrial dynamics proteins of 49 kD (MiD49) and 51 kD (MiD51).^9^, ^10^ During mitochondrial fission, cytosolic Drp1 translocates to the outer mitochondrial membrane (OMM) where it binds to the OMM proteins Mff, MiD49, MiD51 and possibly hFis1, at pre‐constricted sites marked out by contact with endoplasmic reticulum (ER).^11^ Actin polymerization through the ER‐localized inverted formin 2 (INF2) protein has been shown to mediate the mitochondrial pre‐constriction process.^12^, ^13^ Drp1 then oligomerizes and forms a helical structure at the site of pre‐constriction, which encircles and constricts the mitochondrion further, and cleaves the mitochondrion into 2 individual mitochondria in a GTP‐dependent manner.

A number of post‐translational modifications have been shown to regulate Drp1 function in terms of its subcellular localization and protein‐protein interactions. These include phosphorylation, ubiquitination, conjugation of small ubiquitin‐like modifier (SUMO) proteins (SUMOylation), S‐nitrosylation, O‐linked‐N‐acetyl‐glucosamine glycosylation (O‐GlcNAcylation) and most recently acetylation^14^ (reviewed in Ref. 15). Fragmented mitochondria, which have undergone fission, have been visualized by electron microscopy of fixed adult cardiomyocytes in a number of different cardiac diseases including acute myocardial IRI,^16^ myocarditis,^17^ stroke,^18^ doxorubicin cardiotoxicity,^19^ sepsis‐related cardiomyopathy,^20^ post‐AMI cardiomyopathy^21^ and diabetic cardiomyopathy.^22^ In this article, we focus on the role of the mitochondrial fission proteins as potential therapeutic targets for cardioprotection against acute myocardial IRI.

**FIGURE 2:**
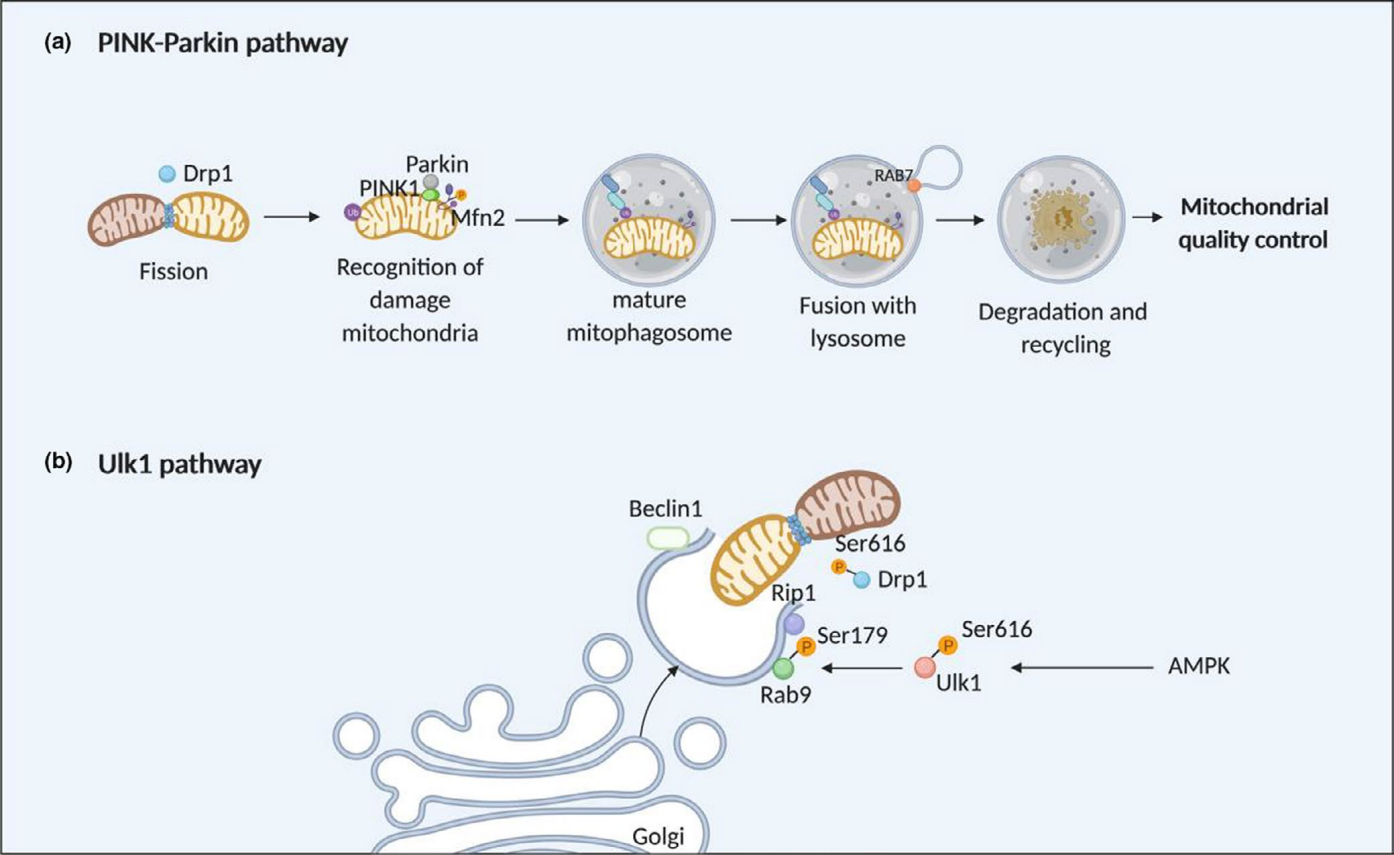
Mitophagy as a target for cardioprotection. The mitochondrial fusion and fission comments have been demonstrated to participate in 2 different mitophagy pathways—(A) the PINK‐Parkin mitophagy pathway and (B) the Ulk1 mitophagy pathway (modified from Ref. 121)

**FIGURE 3:**
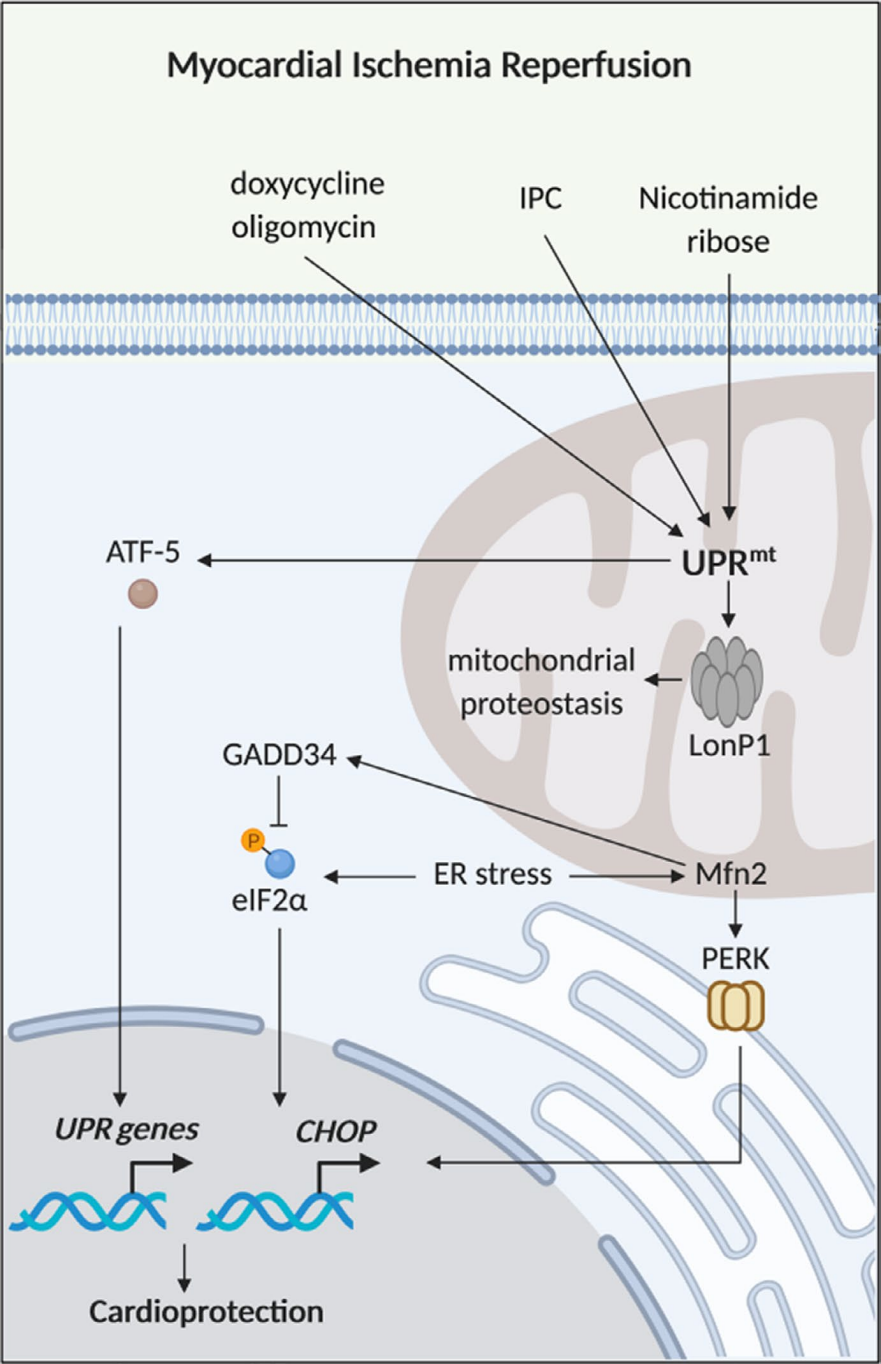
The mitochondrial unfolded protein response and cardioprotection. Schematic model of the mitochondrial unfolded protein response (UPRmt). Nicotinamide ribose, ischaemic preconditioning (IPC), oligomycin and doxycycline are therapeutic strategies that have been shown to reduce acute myocardial ischaemia/reperfusion injury (IRI) by enhancing the UPRmt, and Mfn2 has been shown to play a key role in the UPR

Mitochondrial fusion is characterized by the tethering of two adjacent mitochondria via the OMM fusion GTPase proteins, mitofusin 1 (Mfn1)^23^ and mitofusin 2 (Mfn2),^24^ which then mediate fusion of the OMMs in a GTP‐dependent manner. Subsequently, the inner mitochondrial membrane (IMM) fusion GTPase protein, optic atrophy 1 (OPA1),^25^, ^26^ mediates fusion of the IMMs resulting in sharing of mitochondrial matrix material, and the formation of a single elongated mitochondrion. Importantly, Mfn2 has been reported to have a number of non‐fusion‐related functions including tethering ER to mitochondria,^27^ mediating mitophagy^28^ and cellular autophagy,^29^ and contributing to the stress‐related UPR^30^ (Figures 2 and 3). Similarly, OPA1 has been demonstrated to play several pleiotropic non‐fusion roles related to its effects on cristae remodelling the consequences of which are to regulate mitochondrial cytochrome release^31^ and to improve mitochondrial respiratory efficiency by facilitating the assembly of electron transport supercomplexes.^32^ In addition, OPA1 has been shown to stabilize mitochondrial cristae shape and favour ATP synthase oligomerization and needs ATP synthase to protect mitochondria from stress‐induced respiratory chain inhibition.^33^ Differentiating the pro‐fusion roles of Mfn2 and OPA1 from their non‐fusion roles can be quite challenging and needs to be taken into consideration when investigating their roles in the context of acute myocardial IRI and cardioprotection, where both their pro‐fusion and non‐fusion roles may be important.

A number of post‐translational modifications have been shown to regulate the functions of mitochondrial fusion proteins in terms of their proteins levels and protein‐protein interactions—these include phosphorylation (in Mfn1, Mfn2 and OPA1), acetylation (in Mfn1, Mfn2 and OPA1), methylation (in Mfn1 and OPA1), and ubiquitination (in Mfn1, Mfn2 and OPA1) (reviewed in Ref. 15). The regulation of OPA1 has an added layer of complexity given that there are 8 splice variants (in humans), and proteolytic processing by mitochondrial proteases, YME1L and OMA1, generates several combinations of IMM‐anchored long forms of OPA1 (termed L‐OPA1) which mediate IMM fusion, and soluble short forms of OPA1 within the mitochondrial matrix (termed S‐OPA1), which contribute to mitochondrial bioenergetics and cristae remodelling (reviewed in Ref. 26). Reduced levels of cardiomyocyte Mfn2 and OPA1 have been observed in a number of cardiac diseases including left ventricular hypertrophy,^34^ post‐AMI cardiomyopathy,^21^, ^35^ myocarditis^36^ and diabetic cardiomyopathy.^37^ In this article, we focus on the role of the mitochondrial fusion proteins as potential therapeutic targets for cardioprotection against acute myocardial IRI.

### 2.1 Relevance of mitochondrial fission and fusion proteins to the adult heart

In adult cardiomyocytes, mitochondria are distributed in 3 main locations: intermyofibrillar spaces, where they are densely packed and mainly provide energy for contractile function (interfibrillar mitochondria), just beneath the plasma membrane, where they provide energy for the sarcolemmal ion channels (subsarcolemmal mitochondria), and around the nucleus, where they provide energy for nuclear transcription (perinuclear mitochondria).^38^ Because most adult cardiac mitochondria are fragmented in morphology, and their movement is restricted at these 3 locations, the physiological relevance of mitochondrial morphology and dynamics in the adult cardiomyocyte has been questioned. However, recent studies have shown that the mitochondrial fission and fusion proteins are expressed in the heart and are essential for normal foetal cardiac development, as their genetic ablation is embryonically lethal (reviewed in Refs 2, 3, 4). Post‐natally, they are also required for normal cardiac development and contractile function, as hearts deficient in these proteins develop a severe dilated cardiomyopathy (reviewed in Ref. 2, 3).

Based on the time course over which fusion‐defective mitochondrial size decreases in adult mice deficient in both Mfn1 and Mfn2, it has been estimated that one mitochondrial fusion/fission cycle in adult mouse hearts occurs approximately every 16 days.^39^ More direct evidence of mitochondrial fusion in adult cardiomyocytes has been provided using confocal microscopy imaging of matrix‐targeted photoactivatable green fluorescent protein of whole rat hearts.^40^, ^41^, ^42^ These studies demonstrated rapid content mixing events between adjacent organelles, and slower events between both neighbouring and distant mitochondria, indicative of fusion activity and an interconnected mitochondrial network in adult ventricular cardiomyocytes. Interestingly, mitochondrial fusion activity was shown to decrease with culturing the isolated cardiomyocytes due to a decline in calcium oscillations/contractile activity,^42^ such that the fusion events are slow, with studies showing that the mitochondrial GFP signal takes about 10 hours to spread to the entire population of mitochondria in adult cardiomyocytes.^41^ These findings raise the possibility that mitochondrial fusion may actually occur at much faster rates in the intact contracting heart.

Deletion of cardiomyocyte Drp1 in the early post‐natal period resulted in mitochondrial enlargement, increased mitochondrial interconnectivity, decreased mitochondrial respiration, defective mitophagy and a lethal cardiomyopathy at day 7‐10 of the post‐natal period.^43^, ^44^ Similarly, cardiomyocyte‐specific ablation of Drp1 in adult mice resulted in a severe cardiomyopathy 4 weeks later when compared to wild‐type mice with LV dilatation, reduced LV function, cardiomyocyte hypertrophy, myocardial fibrosis, increased apoptosis, mitochondrial enlargement, impaired mitochondrial respiratory function, increased mitochondrial calcium and ROS, and enhanced susceptibility to mitochondrial permeability transition pore (MPTP) opening.^45^, ^46^, ^47^ Genetic ablation of Mff in mice has been shown to induce a lethal dilated cardiomyopathy at 13 weeks associated with increased heterogeneity in mitochondrial shape and abundance, perturbed mitochondrial respiratory function, increased myocardial apoptosis and interstitial fibrosis.^48^ Interestingly, this effect was reversed if either one of the mitochondrial fusion proteins, Mfn1 or Mfn2, were also deleted suggesting that the pro‐fusion effect of Mff ablation requires the presence of one of the mitofusins. This finding also supports the notion that restoring the balance in mitochondrial fission and fusion in the heart can normalize cardiac function.^48^ With regard to the mitochondrial fission proteins, MiD49 and MiD51, genetic ablation of these proteins is embryonically lethal,^10^ but the effect of their deletion in the adult heart is not known, and needs to be investigated.

With respect to the mitochondrial fusion proteins, cardiomyocyte‐specific deletion of Mfn2 resulted in a mild cardiac phenotype with modest LVH, mild LV systolic dysfunction, no change in mitochondrial respiratory function and unexpected changes in mitochondrial morphology and MPTP opening susceptibility, with pleomorphic and enlarged subsarcolemmal (but not interfibrillar) mitochondria, and resistance to MPTP opening.^49^ Interestingly, the effect of Mfn2 ablation on mitochondrial morphology in neonatal rat cardiomyocytes was as expected, with mitochondrial fragmentation and increased susceptibility to MPTP opening, suggesting cell‐specific effects on mitochondrial morphology and MPTP opening.^49^ In addition, to having enlarged mitochondria, Mfn2‐deficient cardiomyocytes have also being shown by 3D electron microscopy to have fewer mitochondria‐junctional sarcoplasmic reticulum (SR) contacts, and an increase in the distance between mitochondria and junctional SR, when compared to wild‐type cardiomyocytes.^50^ Overexpression of cardiomyocyte‐specific Mfn2 in the adult heart had a very mild phenotype with minor mitochondrial enlargement with no detrimental effects on either mitochondrial respiratory function or LV size or function.^47^ Interestingly, genetic ablation of cardiomyocyte‐specific Mfn1 did result in smaller more fragmented mitochondria, but had no effect on cardiac function or mitochondrial respiratory function, although it did prolong time to induce ROS‐mediated MPTP opening.^51^ The differential effects of Mfn1 versus Mfn2 ablation on mitochondrial size may relate to the pleiotropic non‐fusion effects of Mfn2 such as acting as a tether to the SR.^27^ The mild phenotype observed in hearts deficient in either cardiomyocyte Mfn1 or Mfn2 suggests that these proteins have a redundant function. Genetic ablation of both Mfn1 and Mfn2 mid‐gestation resulted in a severe cardiomyopathy at day 7 of post‐natal period suggesting that the mitofusins are needed for the mitochondrial remodelling which occurs in the first week following birth.^52^ Cardiomyocyte‐specific deletion of both Mfn1 and Mfn2 in the adult heart resulted in mitochondrial fragmentation, impaired mitochondrial respiration, a mitochondrial UFR and a dilated cardiomyopathy.^39^, ^46^, ^50^, ^52^

Although genetic deletion of OPA1 is embryonically lethal, mice with heterozygous ablation of OPA1 survive and have enlarged mitochondria with disorganized cristae, increased time to induce MPTP opening, but had no effect on cardiac function or mitochondrial respiration, although the hearts were more susceptible to LVH induced by total aortic constriction.^53^, ^54^ Heterozygous OPA1 mice did however develop a late‐onset cardiomyopathy associated with fragmented mitochondria with disorganized cristae, impaired mitochondrial respiratory function and increased mitochondrial ROS.^55^ Genetic mouse models with deletions of OMA1 and/or YME1L (the proteases responsible for cleaving L‐OPA1 to S‐OPA1) have been used to investigate the effect of modulating myocardial OPA1 levels on mitochondrial morphology, mitochondrial respiratory function and cardiac function.^56^ Genetic deletion of Yme1l in adult cardiomyocytes was shown to induce mitochondrial fragmentation (due to OMA1 cleaving L‐OPA1 to S‐OPA1), but did not affect mitochondrial respiration, and resulted in dilated cardiomyopathy at 20 weeks.^56^ Interestingly, additional deletion of OMA1 reversed the detrimental effects of YME1L deletion by restoring OPA1 levels.^56^ These studies support an important role for OPA1 in normal cardiac function.

It is interesting and surprising to note that genetic ablation of both the mitochondrial fission protein (Drp1),^16^ and the mitochondrial fusion proteins (Mfn1,^51^ Mfn2^49^ and OPA1^53^, ^54^), had the same effect on MPTP opening in adult cardiac mitochondria, with increased resistance to MPTP opening. This finding suggests that the mechanisms mediating the changes in MPTP opening susceptibility differ between ablation of the fission proteins versus ablation of the fusion proteins. The mechanisms through which genetic ablation of Drp1 reduces the propensity for MPTP opening is not clear, but may relate to the accumulation of factors known to induce MPTP opening such as mitochondrial calcium overload, ROS production and relative ATP depletion. In contrast, it has been postulated that ablation of the outer mitochondrial membrane mitofusin proteins rendered cardiac mitochondria resistant to MPTP opening because of their potential role in OMM remodelling, which may be associated with local perturbations on the integrity of the membrane that predispose to MPTP opening,^49^, ^57^ although further studies are needed to confirm this. The mechanisms through which partial ablation of the inner mitochondrial membrane fusion protein, OPA1, limits MPTP opening susceptibility are not clear, although it has been postulated that partial loss of OPA1 may disturb the organization of the inter‐membrane space and contact points between the outer and inner mitochondrial membranes, and modulate the susceptibility of MPTP opening through this mechanism.^53^, ^54^

In summary, genetic deletion of the mitochondrial fission (Drp1, Mff) or fusion proteins (Mfn2, OPA1) induce changes in mitochondrial morphology, impair mitochondrial respiration and result in dilated cardiomyopathy, confirming that these proteins are essential for normal cardiac development and contractile function.

### 2.2 Potential mechanisms underlying IR‐induced mitochondrial fission

In 2006, Brady et al^58^ made the interesting observation that simulated ischaemia in the HL‐1 cardiac cell line induced a change in mitochondrial morphology from an elongated phenotype to a fragmented one, a change which persisted into simulated reperfusion. Subsequent studies have confirmed that mitochondria undergo Drp‐1‐mediated mitochondrial fission in neonatal and adult rodent hearts.^16^

The mechanisms through which IR induces mitochondrial fission are not clear but a number of factors may play a role: (a) oxidative stress—this is increased in acute myocardial IRI and is known to be an inducer of mitochondrial fission^59^; (b) p38 MAPK—pharmacological inhibition of p38 MAPK at reoxygenation in HL‐1 cells has been shown to reverse hypoxia‐induced mitochondrial fragmentation, suggesting that p38 MAPK activation may in part contribute to hypoxia/reoxygenation‐induced mitochondrial fission^58^; (c) Cdk1 and PKC‐δ—these have been shown to increase during IRI and associate with Drp1, thereby inducing mitochondrial fission^59^; (d) calcium overload—this occurs in IRI and has been shown to induce mitochondrial fission^60^; (e) calcineurin—IRI has been shown to induce calcineurin activation, which is known to dephosphorylate Drp1 at Ser637, thereby promoting translocation of Drp1 to mitochondria to induce fission.^59^, ^61^ Wang et al^61^ have demonstrated that miRNA499 was shown to confer cardioprotection by preventing IR‐induced calcineurin activation and inhibiting Drp1‐mediated fission; (f) SUMOylation of Drp1—it has been reported that Sentrin‐specific protease 3 (SENP3)‐mediated deSUMOylation of Drp1 facilitated its binding to Mff and promoted cell death following simulated IRI^62^, ^63^; (g) succinate—it has been shown that extracellular succinate produced during acute myocardial IRI activated GPR91 and promoted mitochondrial fission by inducing the translocation of Drp1 via PKC‐δ, and the phosphorylation of Mff via Erk1/2^64^; (h) myocardial levels of dual‐specificity protein phosphatase1 (DUSP1), an anti‐apoptotic phosphatase, are reduced following acute myocardial IRI, and it has been shown that mice overexpressing DUSP1 were protected against Mff‐induced mitochondrial fission and BNIP‐mediated mitophagy and had reduced MI size, when compared to control^65^; (i) nuclear receptor subfamily 4 group A member 1 (NR4A1) has been reported to be up‐regulated following acute myocardial IRI and induce mitochondrial fission and suppress mitophagy via casein kinase2 α (CK2α)‐mediated phosphorylation of Mff and FUN14 domain‐containing 1 (FUNDC1), respectively.^66^ Mice deficient in NR4A1 were demonstrated to be protected against acute coronary microvascular injury and mitochondrial dysfunction, when compared with wild‐type mice.^66^

In summary, a number of different factors have been shown to contribute to the observed mitochondrial fission which occurs in response to acute myocardial IRI, thereby providing multiple therapeutic targets for inhibiting mitochondrial fission as a cardioprotective strategy.

### 2.3 Therapeutic targeting of the mitochondrial fission proteins for cardioprotection

It has been shown in HL‐1 cardiac cells that transfection with Drp1 dominant‐negative mutant‐induced mitochondrial elongation prevented MPTP opening and reduced cell death following simulated IRI,^16^ demonstrating Drp‐1 mediated mitochondrial fission to be a therapeutic target for cardioprotection. In this regard, it has been shown that acute inhibition of mitochondrial fission using the putative Drp1 GTPase inhibitor, mitochondrial division inhibitor 1 (mdivi‐1), reduced cell death in isolated adult murine cardiomyocytes subjected to simulated IRI and reduced MI size in murine hearts subjected to in vivo acute myocardial IRI.^16^ Mdivi‐1 has also been shown to inhibit IR‐induced mitochondrial fragmentation in a diabetic mouse model of acute myocardial IRI^67^ and inhibited mitochondrial fission and restored the cardioprotective effects of sevoflurane under conditions of high glucose in neonatal rat cardiomyocytes.^68^ It has also been demonstrated that mdivi‐1 reduced cell death in human W8B2^+^ cardiac stem cells subjected to simulated IRI, although in that study no beneficial effects were observed on mitochondrial morphology so the mechanisms underlying the cardioprotective effect in these cells are unclear.^69^ Other pharmacological inhibitors of mitochondrial fission have also been shown to be cardioprotective including Dynasore (a non‐specific inhibitor of dynamins),^70^ and P110, a peptide inhibitor that inhibits the interaction between Drp1 and hFis.^71^ Importantly, pharmacological inhibition of mitochondrial fission at the onset of reperfusion, a clinically relevant time‐point, using either P110^71^ or mdivi‐1^72^ was shown to reduce MI size in small animal models of acute myocardial IRI. In Table 1, we present a summary of the main experimental studies investigating cardioprotection with pharmacological agents targeting mitochondrial fission proteins.

**TABLE 1:** Summary of major studies investigating cardioprotection with pharmacological agents targeting mitochondrial fission proteins

Mitochondrial fission and fusion protein	Pharmacological agent	Animal acute myocardial IRI models	Cardioprotective effect	Comment
Drp1^16^	Mdivi‐1, pre‐treatment	In vivo mouse heart	Reduced MI size and less fission	
Drp1^67^	Mdivi‐1, 15 min prior to reperfusion	In vivo mouse heart	Reduced MI size, less fission, improved mitochondrial function	
Drp1^81^	Mdivi‐1, alone or in nanoparticles at reperfusion	Ex vivo mouse heart	Both mdivi‐1 alone or in nanoparticles reduced MI size	Mdivi‐1 delivered by nanoparticles more cardioprotective.
Drp1^81^	Mdivi‐1, alone or in nanoparticles at reperfusion	In vivo mouse heart	Mdivi‐1 only reduced MI size when delivered by nanoparticles	Mdivi‐1 delivered by nanoparticles more cardioprotective.
Drp1^80^	Mdivi‐1, at reperfusion	In vivo closed‐chest pig heart	No reduction in MI size or change in mitochondrial morphology	Underpowered study.
Drp1^70^	Dynasore, during reperfusion	Ex vivo mouse heart	Reduced MI size	
hFis1‐Drp1 interaction^71^	P110 in stabilization and during reperfusion	Ex vivo rat heart	Reduced MI size	
hFis1‐Drp1 interaction^71^	P110 at reperfusion	In vivo rat heart	Reduced MI size	
Drp1^75^	Drpitor1 and Drpitor1a pre‐treatment	Ex vivo rat heart	Improved right ventricle function, less fission and mitochondrial ROS production	

Abbreviation: MI, myocardial infarct.

Recent studies suggest that mdivi‐1 has off‐target effects that are independent of its inhibitory effects on Drp1 GTPase activity, including it being a weak and reversible inhibitor of complex I, and modulating mitochondrial production of ROS in neurons,^73^ and inhibiting complex II, and modulating transient opening of the MPTP in isolated adult cardiomyocytes.^74^ Novel, more specific inhibitors of Drp1 itself or its interaction with its OMM receptors, Mff and MiD49/MiD51, would provide more effective cardioprotection and facilitate the translation of acute mitochondrial fission inhibition as a cardioprotective strategy. In this regard, Wu et al^75^ have identified 2 novel Drp1 inhibitors (Drpitor1 and Drpitor1a) which have been shown to be more potent and specific than mdivi‐1 in terms of inhibiting Drp1 GTPase activity (without complex I inhibition), and which conferred cardioprotection against acute IRI in the isolated perfused rat heart when given as a pre‐treatment.

Genetic inhibition of Drp1 using either siRNA^61^ or Drp1 dominant‐negative K38A mutant^76^ has been shown to prevent translocation of Drp1 to mitochondria, reduce cell death in isolated rat cardiomyocytes subjected to IRI and reduce MI size in rat hearts subjected to in vivo acute IRI. With respect to the other mitochondrial fission protein, Mff, it has been reported that Mff levels increase in neonatal rat cardiomyocytes subjected to hydrogen peroxide,^77^ and in murine hearts subjected to acute IRI.^78^ Importantly, siRNA ablation of Mff prevented hydrogen peroxide‐induced mitochondrial fission, and siRNA knockdown of Mff also reduced MI size in the adult mouse heart subjected to acute IRI.^77^ In addition, adult mice deficient in Mff (by gene trap) have been reported to have smaller MI size, and better cardiac function compared to wild‐type mice.^78^ The role of the mitochondrial fission proteins, MiD49 and MiD51 has not been studied as potential targets for cardioprotection, although they have been reported together with Drp1 to mediate mitochondrial division that is needed for vascular smooth muscle cell proliferation in pulmonary arterial hypertension (PAH) animal models,^79^ positioning mitochondrial fission as a therapeutic target for PAH.

Taken together, these findings demonstrate mitochondrial fission to be a target for cardioprotection in rodent models of acute myocardial IRI, and the next step in the translational pathway is to test the efficacy of this therapeutic approach in a large animal AMI model. In this regard, a recent small pilot study has tested the cardioprotective effects of mdivi‐1 administered as an intracoronary bolus immediately prior to reperfusion in the clinically relevant closed‐chest pig AMI model. However, it found no difference in MI size, LV systolic function, myocardial fibrosis or mitochondrial morphology after 3 days reperfusion when compared to vehicle control.^80^ Potential reasons for the lack of cardioprotection include the small sample size, potential off‐target effects of mdivi‐1 and insufficient delivery of mdivi‐1 into the ischaemic myocardium in the first few minutes of reperfusion. In terms of the latter issue, it has been shown that nanoparticle delivery of mdivi‐1 to the ischaemic heart enhanced its cardioprotective effect in terms of MI size reduction in the murine AMI model,^81^ and nanoparticle delivery of mdivi‐1 to the ischaemic heart may be more effective in the pig AMI model. Furthermore, it would be interesting to investigate whether more specific inhibitors of the mitochondrial fission machinery such as P110^71^ may be more effective in this large animal AMI model.

The beneficial effects of inhibiting mitochondrial fission have also been investigated in the coronary microvasculature following acute myocardial IRI, a neglected target for cardioprotection.^82^ Melatonin, a well‐established cardioprotective factor, has been shown to reduce coronary microvascular injury, as evidenced by preserved endothelial integrity, increased endothelial nitric oxide synthase (eNOS) expression, less lumen obstruction, attenuated inflammatory cell infiltration and less endothelial cell death, findings which were associated with reduced mitochondrial fission and less MPTP opening in coronary endothelial cells.^83^ Similarly, the sodium‐glucose cotransporter 2 (SGLT2) inhibitor, empagliflozin^84^ and Bax inhibitor 1 (BI1)^85^ have been reported to reduce coronary microvascular injury by inhibiting mitochondrial fission via the Syk–Nox2‐Drp1 signalling pathway. Finally, mice deficient in Mff have been reported to be protected against IRI to the coronary microvasculature an effect which was attributed to less mitochondrial fission, attenuated cardiolipin oxidation and apoptosis and inhibition of MPTP opening (via blocking the oligomerization of VDAC1 and HK‐2 separation from mitochondria) in coronary microcirculation endothelial cells.^78^ These findings support the notion that therapeutic inhibition of mitochondrial fission may accrue dual benefits against acute myocardial IRI with cytoprotective effects on both cardiomyocytes and the coronary microvasculature. In other organs, such as kidney^86^ and brain,^18^ acute IRI has also been shown to induce mitochondrial fragmentation, with genetic and pharmacological inhibition of Drp1 also conferring protection.

In summary, experimental small animal studies have provided convincing evidence that acute inhibition of mitochondrial fission is cardioprotective, and novel more specific inhibitors of the fission machinery are needed to translate this therapeutic strategy for patient benefit. However, although *acute* inhibition of mitochondrial fission has been shown to be cardioprotective, *chronic* inhibition of fission is known to be detrimental, with cardiomyocyte‐specific genetic deletion of Drp1^45^ increasing susceptibility to acute myocardial IRI, an observation which most likely relates to the suppression of mitophagy, resulting in accumulation of damaged mitochondria and the development of cardiomyopathy (see Section 3). These findings underscore the importance of balancing mitochondrial fusion and fission for normal cardiac function.

### 2.4 Other cardioprotective interventions targeting IR‐induced mitochondrial fission

A number of established cardioprotective interventions, treatments and factors have been shown to mediate their beneficial effects via inhibition of mitochondrial fission and preservation of mitochondrial function, following acute myocardial IRI. These include (a) aerobic interval training^87^; (b) nitrite pre‐treatment, which has been shown to activate PKA which in turn phosphorylates Drp1 at Ser637, thereby preventing IR‐induced mitochondrial fission^88^; (c) chronic therapy with the SGLT2 inhibitors, either empagliflozin^89^ or dapagliflozin,^90^ therapies which have been shown to improve cardiovascular outcomes, have been demonstrated to inhibit mitochondrial fission and reduce MI size in rodent models of acute myocardial IRI; (d) zinc, a known cardioprotective agent, was reported to mediate SUMOylation of Drp1 thereby inhibiting mitochondrial fission and reducing MI size in the mouse heart^91^; (e) proto‐oncogene serine/threonine protein kinase (PIM‐1) has been shown to reduce mitochondrial translocation of Drp1 and inhibit mitochondrial fission following acute myocardial IRI^92^; (f) vagal nerve stimulation which is a known cardioprotective strategy has been shown to inhibit mitochondrial fission, preserve mitochondrial function and prevent MPTP opening in acute myocardial IRI small animal models^93^, ^94^; (g) Parkinson's disease‐related mitochondrial protein, DJ‐1^95^ has been shown to protect the heart by regulating the SUMOylation status of Drp1, thereby attenuating IR‐induced mitochondrial fission^96^; (h) ginsenoside Rg5 has been shown to activate Akt and mitochondrial hexokinase II, thereby attenuating the translocation of Drp‐1 to mitochondria and inhibiting fission in an isoproterenol (ISO)‐induced acute myocardial IRI mouse model^97^; (i) isosteviol sodium, a component of artificial sweetners, has been shown to inhibit mitochondrial fission and reduce MI size in the isolated guinea pig heart subjected to acute IRI^98^; (j) miRNA763 has been demonstrated to inhibit Mff‐induced mitochondrial fission and reduced MI size in a mouse acute IRI model^77^; and finally (k) propofol has been reported to inhibit mitochondrial fission and reduce cell death in H9C2 cells subjected to simulated IRI.^99^

In summary, these studies implicate IR‐induced mitochondrial fission to be a critical mediator of cardiomyocyte death following AMI that can be targeted indirectly via a wide variety of cardioprotective agents.

### 2.5 Targeting mitochondrial fusion proteins for cardioprotection

Given the data supporting mitochondrial fission to be a key determinant of cardiomyocyte death following acute IRI, studies have explored the effect of promoting mitochondrial fusion as a target for cardioprotection. However, as mentioned previously, the interpretation of these studies can be quite challenging given the pleiotropic non‐fusion functions of Mfn2 and OPA1, which may also contribute to cardioprotection. An early study in COS‐7 cells demonstrated that activating Mfn2 protected cells against apoptotic cell death and ROS‐induced MPTP opening.^100^ In contrast to this study, it has been shown that overexpressing Mfn2 in neonatal cardiomyocytes increased apoptotic cell death.^101^ In HL‐1 cardiac cells, it has been reported that overexpressing either Mfn1 or Mfn2 prevented IR‐induced mitochondrial fragmentation, inhibited MPTP opening and reduced cell death following simulated IRI.^16^ In addition, activation of the cardioprotective kinase, Akt, by either genetic activation or erythropoietin has been shown to induce mitochondrial elongation via Mfn1, inhibit MPTP opening and protect the heart against acute IRI.^102^

In the adult heart, the effects of modulating cardiomyocyte mitofusins have produced unexpected effects in terms of susceptibility to acute IRI. Adult mouse studies have reported that isolated cardiomyocytes lacking Mfn1 displayed mitochondrial fragmentation and were protected against hydrogen peroxide‐induced MPTP opening and cell death.^51^ Similarly, cardiomyocyte‐specific deletion of Mfn2 in the adult heart induced a modest LVH associated with pleomorphic enlargement of mitochondria, and inhibition of MPTP opening, and increased resistance to acute myocardial IRI.^49^ The mechanisms underlying the cardioprotective effects of individual Mfn1 and Mfn2 cardiomyocyte ablation were not clear from these studies. Interestingly, it has been shown that deletion of both Mfn1 and Mfn2 in the adult heart inhibited MPTP opening and reduced MI size,^50^ a cardioprotective effect which was attributed to the pleiotropic non‐fusion role of Mfn2 in tethering mitochondria to the sarcoplasmic reticulum to create localized microdomains through which calcium can transit.^27^, ^103^ Depletion of cardiomyocyte mitofusins was demonstrated to confer cardioprotection by reducing tethering of mitochondria to SR and attenuating IR‐induced mitochondrial calcium overload and MPTP opening.^50^ Therefore, targeting Mfn2 during acute myocardial IRI to transiently disassociate mitochondria from SR may provide a novel cardioprotective strategy. In this regard, Franco et al^104^ have engineered cell‐permeant mini‐peptides with the ability to destabilize the fusion‐constrained conformation of mitofusin (thereby inhibiting Mfn2) and promote the fusion‐permissive conformation (thereby activating Mfn2), but their effect on mitochondrial morphology and susceptibility to acute IRI in the adult heart remains to be tested.

With respect to the mitochondrial fusion protein, OPA1, studies have investigated its role as a cardioprotective target, but as with Mfn2, attributing the cardioprotective effects of OPA1 to its pro‐fusion function has been challenging, given its potential cardioprotective non‐fusion effects on mitochondrial cristae remodelling that prevent apoptosis and facilitate the assembly of electron transport supercomplexes and improve mitochondrial respiratory efficiency. Chen et al^35^ showed reduced myocardial levels of OPA1 and mitochondrial fragmentation in both rat and human models of heart failure, and demonstrated in H9C2 cells that simulated IR reduced levels of OPA1, and siRNA ablation of OPA1 induced mitochondrial fragmentation, and increased susceptibility to IRI. Recently, it has been shown in human atrial tissue obtained from patients undergoing cardiac bypass surgery that myocardial L‐OPA1 levels were decreased and this was associated with mitophagy and mitochondrial biogenesis from the IRI stress of heart surgery.^105^ Le Page et al^54^ have shown that OPA1± mice (which have a 30% reduction in OPA1 levels) were more susceptible to acute myocardial IRI, although this effect was not associated with changes in mitochondrial morphology, mitochondrial respiratory function, apoptosis or MPTP opening, and so the mechanisms underlying this cardioprotective effect are not clear from this study. In contrast, it has been shown in the adult mouse that genetic overexpression of OPA1 stabilized mitochondrial cristae thereby improving mitochondrial respiratory efficiency, preventing mitochondrial dysfunction, attenuating cytochrome c release and ROS production, and through these actions, it reduced susceptibility to acute myocardial IRI.^106^ However, the effect of OPA1 overexpression on preventing IR‐induced mitochondrial fragmentation was not assessed, as the cardioprotective effects of OPA1 were attributed to its non‐fusion effects on mitochondrial cristae remodelling and improving mitochondrial respiratory efficiency. Importantly, in this mouse model of OPA1 overexpression, there were beneficial effects in other organs with protection from muscular atrophy, brain ischaemia and liver apoptosis.^106^

Interestingly, the role of OPA1 as a cardioprotective target has been investigated in the context of remote ischaemic conditioning (RIC), an endogenous cardioprotective strategy in which cycles of brief non‐lethal ischaemia and reperfusion applied to the limb increase resistance to a sustained episode of lethal acute myocardial IRI.^107^, ^108^, ^109^ These authors showed that using a tourniquet to induce four‐ to 5‐minute cycles of hindlimb ischaemia and reperfusion reduced MI size, and this was associated with less mitochondrial fragmentation and preservation of myocardial OPA1 levels without affecting levels of Mfn2.^109^ Further studies are needed to investigate whether RIC increased myocardial OPA1 levels by suppressing stress‐induced activation of OMA1. The cardioprotective agent, melatonin, has recently been demonstrated to confer cardioprotection via AMPK‐OPA1 as evidenced by reduced MI size, inhibition of IR‐induced mitochondrial fission, preservation of mitochondrial respiratory function, less MPTP opening and decreased apoptosis.^110^ Similarly, the brain‐derived neurotrophic factor mimetic, 7,8‐dihydroxyflavone, has been reported to confer cardioprotection via inhibition of IR‐induced mitochondrial fission and decreased apoptosis.^111^ Finally, modulation of OPA1 levels by genetic ablation of OMA1 has also been shown to protect against acute renal IR acute injury.^112^ In terms of therapeutic targeting of OPA1 as a cardioprotective strategy, a recent study has identified epigallocatechin gallate to be a novel pharmacological inhibitor of OMA1. Treatment of mouse embryonic fibroblasts was able to prevent cleavage of L‐OPA1 to S‐OPA1, inhibit mitochondrial fission, prevent apoptosis and reduce cell death following simulated IRI, providing a potential therapeutic strategy for reducing MI size following acute myocardial IRI.

In summary, these studies implicate the mitochondrial fusion proteins, Mfn2 and OPA1, to be potential targets for cardioprotection, but the mechanisms underlying their beneficial effects appear to be related to their non‐fusion pleiotropic functions rather than their pro‐fusion effects.

## 3 TARGETING MITOPHAGY FOR CARDIOPROTECTION

Under basal conditions, mitophagy is required for mitochondrial quality control and plays a key role in removal of damaged mitochondria.^113^ Mitophagy is activated during acute myocardial IRI, where it appears to protect the heart against cell death, as it preserves energy substrates, removes damaged mitochondria and attenuates oxidative stress. In this regard, it has been shown that inhibition of mitophagy in mice deficient in cardiomyocyte Drp1^45^ or PGAM5^114^ or treatment with bicarbonate^115^ increased cell death following acute IRI. In contrast, activation of mitophagy prior to acute myocardial ischaemia, by cardioprotective strategies such as ischaemic preconditioning^116^ and simvastatin,^117^ has been shown to reduce myocardial infarction size following acute myocardial IRI. It has also been shown that mitophagy activation occurs in human heart tissue harvested from patients subjected to acute myocardial IRI during cardiac surgery and is associated with increases in mitochondrial biogenesis, where it promotes turnover of cardiac mitochondria.^105^

The canonical PINK‐Parkin pathway for homeostatic mitophagic mitochondrial quality control was originally defined by genetic manipulation of these two factors in *Drosophila* fruit flies,^118^, ^119^ establishing the mitophagy mechanistic link between the mitochondria‐localized kinase, PINK and the downstream cytosolic E3‐ubiquitin ligase, Parkin (Figure 2). Contrasting with the situation in normal hearts wherein Parkin is barely detectable and functionally dispensable, Parkin is transcriptionally up‐regulated in different forms of cardiac stress where it plays an important role in the mitophagic response.^46^, ^120^, ^121^ Compared to wild‐type mice, Parkin‐deficient mice had disorganized mitochondrial networks and smaller mitochondria, although mitochondrial and cardiac function were unaffected.^120^ However, the Parkin‐/‐ mice were more susceptible to acute myocardial IRI and had reduced survival and mitophagy with accumulated swollen, dysfunctional mitochondria after  infarction.^120^ In contrast, overexpression of Parkin in isolated cardiomyocytes protected against simulated ischemic injury.^120^ A cardioprotective role for PINK1 kinase has also been observed in a mouse AMI model.^122^ These data suggest cardioprotective effects of the PINK‐Parkin mitophagy pathway in the setting of acute myocardial IRI.

Drp1‐mediated mitochondrial fission generates fragmented mitochondria for selective removal by Parkin‐induced and Bnip3‐induced mitophagy^123^, ^124^ as a key strategy for maintaining mitochondrial quality control. It has been shown that siRNA ablation of Drp1 in neonatal ventricular rat cardiomyocytes induced mitochondrial elongation, increased apoptosis, suppressed autophagic flux, resulting in accumulation of dysfunctional mitochondria and increased MPTP opening.^45^ Overexpression of Drp1 in cardiomyocytes induced mitochondrial fragmentation, enhanced autophagic flux and increased apoptotic cell death.^45^ Cardiomyocyte‐specific ablation of Drp1 in adult mice at 12 weeks of age induced cardiomyocyte hypertrophy and a lethal dilated cardiomyopathy with mitochondrial elongation, increased apoptosis, suppressed autophagic flux, accumulation of dysfunctional mitochondria and increased MPTP opening.^45^ Interestingly, both heterozygous Drp1 mice and mice with cardiomyocyte‐specific ablation of Drp1 in adult mice increased MI size, suggesting that chronic Drp1 ablation and perturbed mitophagy increases susceptibility to acute myocardial IRI,^45^ which differs from acute inhibition of mitochondrial fission which has been shown to be cardioprotective.^16^

Mfn2 involvement in mitophagy is contextually complex, recruiting Parkin to mitochondria for normal perinatal metabolic remodelling,^28^, ^125^ controlling mitochondrial quantity in normal adult hearts^46^, ^126^ and mediating stress‐induced calcium signalling between cardiomyocyte ER and mitochondria.^127^, ^128^, ^129^ How these different mechanisms of mitofusin‐involved mitophagy overlap, complement or compete with each other and with other mitophagy pathways remains incompletely understood. Interestingly, Mfn2 has also be reported to be required for cardiac autophagy, by potentially functioning as an adaptor on ER‐derived autophagosome membrane for RAB7, a autophagosome maturation‐related protein, to mediate the fusion of autophagosomes with lysosomes.^29^ Mice deficient in cardiomyocyte Mfn2 were shown to accumulate autophagosomes due to impaired fusion of autophagosomes with lysosomes, resulting in impaired mitochondrial function, slightly enlarged mitochondria and increased susceptibility to acute myocardial IRI,^29^ suggesting an important role for Mfn2‐mediated autophagy as a cardioprotective target.

Recently, a novel PINK/Parkin‐independent pathway of mitophagy has been described which functions under conditions of energy stress including starvation and acute myocardial IRI^130^ (Figure 2). This is based on a protein complex consisting of unc‐51‐like kinase 1 (Ulk1), Rab9, receptor‐interacting serine/threonine protein kinase 1 (RIP1) and Drp1, which allows the recruitment of trans‐Golgi membranes associated with Rab9 to damaged mitochondria through phosphorylation of Rab9 at S179 by Ulk1 and phosphorylation of Drp1 at S616 by RIP1.^130^ This novel Ulk1/Rab9/Rip1/Drp1 mitophagy pathway was shown to protect the heart against acute myocardial IRI by maintaining healthy mitochondria.^130^

In summary, enhancing mitophagy during acute myocardial IRI is an important strategy for cardioprotection. Given the close interplay between mitochondrial morphology and mitophagy, therapeutic targeting of the mitochondrial fission and fusion proteins may provide a strategy for boosting mitophagy following AMI.

## 4 TARGETING THE MITOCHONDRIAL UNFOLDED PROTEIN RESPONSE FOR CARDIOPROTECTION

The mitochondrial UPR (UPR^mt^) is a cytoprotective signalling pathway triggered by the mitochondrial accumulation of toxic unfolded proteins under conditions of cellular stress that induces mitochondrial dysfunction (Figure 3). The UPR^mt^, in turn, up‐regulates cyclic AMP‐dependent transcription factor ATF‐5 (ATF5) to restore mitochondrial proteostasis and respiratory function.^131^ The mitochondrial fusion protein, Mfn2, which is known to tether mitochondria to the ER, has been shown to regulate the UPR. Ngoh et al^30^ showed that ER stress induced the gene and protein expression of Mfn2 but not Mfn1, with ablation of Mfn2 (but not Mfn1) in adult murine cardiomyocytes exacerbating ER stress‐mediated apoptotic cell death. It has been shown that Mfn2 regulates the UPR and mitochondrial respiratory function by down‐regulating protein kinase RNA‐like endoplasmic reticulum kinase (PERK), an ER stress‐related protein.^132^ Adult mice deficient in cardiomyocyte Mfn2 have also been shown to have increased ER stress (as evidenced by increased expression of ER stress‐related genes Asns, Atf4, Trib3, Chop, ATF6, and Gsta1), supporting a role for Mfn2 in preventing ER stress in the adult heart.^29^ The role Mfn2 plays specifically in UPR^mt^ remains to be established. The UPR^mt^ has recently been demonstrated to occur in heart diseases such as cardiac failure,^133^ pressure overload^134^ and acute myocardial IRI,^5^ making it an attractive therapeutic target for cardioprotection.

It has been shown in mice hearts subjected to pressure‐overload hypertrophy and in hypertrophied human heart tissue due to aortic stenosis, that the UPR^mt^ is increased as evidenced by up‐regulation of ATF5 and the C/EBP transcription factor CCAT‐enhancer‐binding protein homologous protein (CHOP), which encodes adaptive proteins of the UPR^mt^.^134^ Importantly, from a therapeutic perspective, it was reported that the UPR^mt^ could be enhanced using nicotinamide riboside (which augments NAD^+^ pools) in cardiomyocytes and prevented cardiomyocyte death and preserved mitochondrial respiratory function and cardiac contractile function.^134^ Pharmacological induction of the UPR^mt^ using either oligomycin or doxycycline reduced MI size in wild‐type mice but not ATF5‐deficient mice, suggesting that ATF5‐mediated UPR^mt^ confers cardioprotection against acute IRI.^5^ The mitochondrial protease, LonP1, which ensures mitochondrial proteostasis and regulates adaptive responses to cell stress, has been shown to contribute to the cardioprotection elicited by ischaemic preconditioning (IPC), an endogenous cardioprotective intervention in which brief cycles of non‐lethal ischaemia and reperfusion protect the heart against an episode of acute lethal IRI.^135^ IPC was shown to up‐regulate LONP1 in wild‐type mice, and heterozygous LONP1± mice were shown to be more susceptible to acute myocardial IRI as evidenced by mitochondrial dysfunction and increased MI size, and were found to be resistant to IPC‐induced cardioprotection.^136^ In contrast, genetic overexpression of cardiomyocyte‐specific LONP1 prevented IR‐induced mitochondrial dysfunction and reduced MI size, implicating mitochondrial LONP1 as an endogenous mediator of cardioprotection.^136^

In summary, enhancing the UPR^mt^ during acute myocardial IRI has emerged as a novel therapeutic cardioprotective strategy. Although pharmacological strategies such as nicotinamide riboside, oligomycin and doxycycline have been shown to confer cardioprotection by enhancing the UPR^mt^, novel more specific activators of the UPR^mt^ are needed.

## 5 CONCLUSIONS

Preventing mitochondrial dysfunction during acute myocardial IRI following AMI is a major therapeutic strategy for cardioprotection in terms of reducing MI size, preserving cardiac function and preventing the onset of HF. In this regard, the mitochondrial fission and fusion proteins which play key roles in process involved in mitochondrial quality control (such as mitochondrial morphology, mitophagy and UPR^mt^), act to preserve normal mitochondrial respiratory function in the setting of acute myocardial IRI, positioning them as key targets of cardioprotection. Substantial experimental data suggest that acute inhibition of mitochondrial fission can reduce MI size and preserve cardiac function in small animal models of AMI. Further studies are needed to test this therapeutic approach in clinically relevant large animal AMI models and to discover novel more specific inhibitors of the mitochondrial fission machinery. However, it is important to note that chronic inhibition of mitochondrial fission is detrimental to both susceptibility to acute IRI and cardiac function, as it suppresses mitophagy and results in the accumulation of damaged mitochondria. Although the mitochondrial fusion protein, OPA1, has been shown to be a target for acute cardioprotection (with its overexpression protecting against acute myocardial IRI), its beneficial effects have been attributed to non‐fusion pleiotropic roles rather than its effects on mitochondrial morphology. The role of the mitofusins as targets for acute cardioprotection has been unexpected, as it appears that ablation of Mfn1 and/or Mfn2 confers a protective response against acute myocardial IRI, and again this may be due to their non‐fusion pleiotropic roles (such as tethering SR), rather than their effects on mitochondrial morphology. Overall, targeting the mitochondrial fusion and fission proteins have emerged as important targets for cardioprotection and have the therapeutic potential to reduce MI size, preserve cardiac function and prevent HF in patients presenting with AMI.

## CONFLICT OF INTEREST

The authors confirm that there are no conflicts of interest.

## AUTHOR CONTRIBUTION

SRH, HG, FP, DJH, GD, all contributed to the design, writing and critical reading of the paper.
